# A computational cognitive model of self-efficacy and daily adherence in mHealth

**DOI:** 10.1007/s13142-016-0391-y

**Published:** 2016-02-22

**Authors:** Peter Pirolli

**Affiliations:** Palo Alto Research Center, Palo Alto, CA 94304 USA

**Keywords:** ACT-R, Self-efficacy, Adherence, Computational model

## Abstract

Mobile health (mHealth) applications provide an excellent opportunity for collecting rich, fine-grained data necessary for understanding and predicting day-to-day health behavior change dynamics. A computational predictive model (ACT-R-DStress) is presented and fit to individual daily adherence in 28-day mHealth exercise programs. The ACT-R-DStress model refines the psychological construct of *self-efficacy*. To explain and predict the dynamics of self-efficacy and predict individual performance of targeted behaviors, the self-efficacy construct is implemented as a theory-based neurocognitive simulation of the interaction of behavioral goals, memories of past experiences, and behavioral performance.

## A COMPUTATIONAL COGNITIVE MODEL OF SELF-EFFICACY AND DAILY ADHERENCE IN mHEALTH

There is a pressing need to extend the reach of existing health behavior change programs in areas such as diet, fitness, and stress and to intensify and prolong their impact. Mobile health (mHealth) platforms provide an excellent opportunity for projecting supportive motivational, cognitive, and social interventions for behavior change into everyday life at great economies of scale. Especially promising is the opportunity for precisely measuring the dynamics of psychosocial factors as people participate in mHealth programs and, based on those assessments, providing personalized interactions that optimize desirable achievements. The challenge posed by these opportunities for detailed measurement and intervention is that current theorizing and modeling of individual health behavior change is not equally fine-grained and predictive [1, 2].

In this paper, I present a computational model, called ACT-R-DStress, of individual daily adherence data from a study [3] of an mHealth app, called DStress. The DStress app provides personalized exercise and meditation goals that can adjust in difficulty based on past adherence. The ACT-R-DStress model refines the psychological construct of *self-efficacy* [4] that is core to social cognitive theory and conceptually equivalent to the construct of *perceived behavioral control* (PBC) in the theory of planned behavior [5]. Self-efficacy is an individual’s belief that he or she is capable of performing a behavioral goal. In general, the higher the level of self-efficacy, the greater the confidence in one’s ability to succeed at a goal and the greater the likelihood of achieving the goal. The model also refines a construct of *intended effort* proposed by Kukla [6, 7] that modulates intensity of goal striving based on differences between self-efficacy and the perceived difficulty of a goal. Levels of self-efficacy and goal striving effort are often strong predictors of success in programs aimed at changing behavior in a wide range of areas [8].

ACT-R-DStress is instantiated as simulations in the ACT-R neurocognitive architecture [9, 10]. ACT-R is a theory of how the functions of the mind arise from the structure of the brain. ACT-R is also a computational architecture for simulating and understanding learning and cognition. More generally, ACT-R explains how the mind organizes knowledge and experience to produce behavior. The ACT-R-DStress simulations are used to predict each individual’s success in performing each assigned exercise in a controlled 28-day study reported in Konrad et al. [3], in which the difficulty of exercise goals varied from day to day. In addition to the detailed ACT-R-DStress simulation model, I also present a mathematical model based on ACT-R-DStress that also provides good fits to the daily adherence data but without the need for full-scale ACT-R-DStress simulation. These models can be used to predict the likelihood of a given person performing behavioral goals of varying difficulty, which could be useful in tailoring daily goals in mHealth apps to maximize expected gains.

## BACKGROUND

### The DStress system

DStress [3] is a web- and mobile-based system that provides a simple form of automated coaching on exercise and meditation goals aimed at reducing perceived stress. The coaching algorithms modulate the difficulty of daily exercise and meditation goals based on individuals’ performance on the immediately preceding goals. An overarching aim of the automated coaching is to progressively increase the difficulty of exercise and meditation goals. Over the course of several weeks, individuals can achieve goals that they could not do at the beginning of the program.

**Fig. 1:**
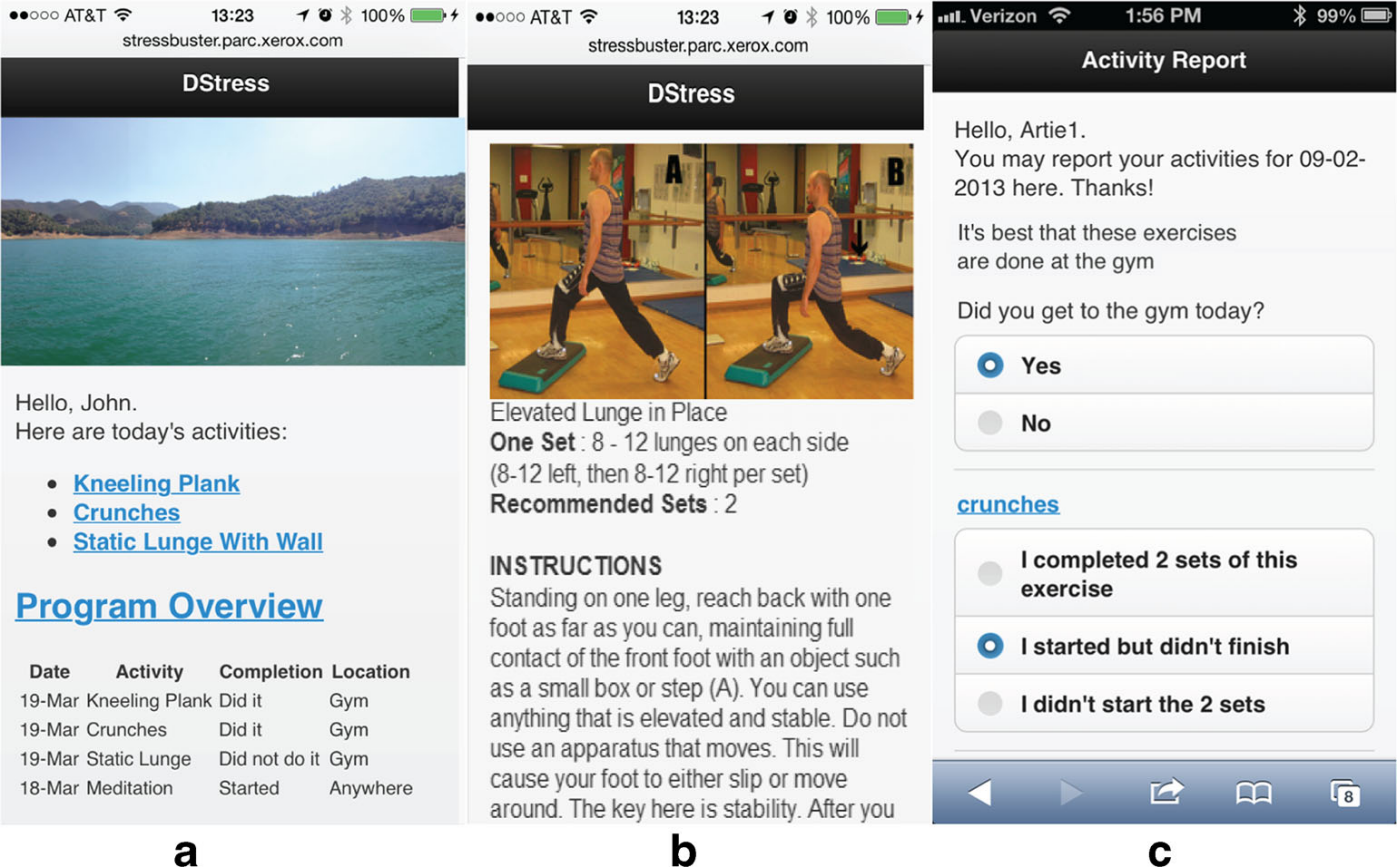
The DStress application for reducing stress: (**a**) the home screen showing daily goals and part adherence, (**b**) instruction screen, and (**c**) reporting screen

Figure 1 presents several screenshots from the DStress system. Over the course of a multi-week program, users are sent an email every morning with a reminder to login to DStress. On the DStress homescreen (Fig. 1a), users are presented with their current goals, as well as previous activities and their completion status. Clicking on any activity takes the user to pictures and detailed instructions of how to safely and properly perform each activity (Fig. 1b). Users can also click on an activity to report whether or not they performed their goal for the day (Fig. 1c), and users are sent an email reminder in the evening if they fail to report.

The current DStress programs have three kinds of days: exercise days (occurring on Mondays, Wednesdays, and Fridays), meditation days (Tuesdays, Thursdays, and Saturdays), and rest days (Sundays). Three certified personal trainers developed a pool of 46 exercises such as wall push-ups, standing knee lifts, squats, and burpees and also rated the difficulty of the exercises. The personal trainers proposed the exercises for a target population of adults interested in reducing stress.

The details of the DStress coaching heuristics for progressing people to more difficult goals or regressing them to easier goals are described in detail in Konrad et al. [3]. Generally, if a person successfully completes all exercises assigned for a day, they advance to the next level. If they do not succeed at exercises or meditation activities, then they are regressed to exercises or meditation activities at an easier level.

### Self-efficacy and enactive mastery

The rationale for the automated personalization of goal difficulties in DStress can be derived from several social-psychological and individual-health theories, including goal-setting theory [11], social cognitive theory [4], and the theory of planned behavior [5]. Goal-setting theory predicts that goals need to be challenging enough to be motivating. However, the construct of self-efficacy in social cognitive theory (or PBC in the theory of planned behavior) predicts that goals that are perceived as too difficult are unlikely to be attempted. So, one challenge for coaching (automated or not) is to provide goals that are difficult enough to be motivating but easy enough to be successfully achieved.

In addition to selecting specific daily goals that achieve high success rates, there is the problem of increasing individuals’ levels of self-efficacy so that more difficult goals can be achieved in the future. This can be achieved through *guided enactive mastery*, in which individuals are supported in achieving progressively difficult goals: “Enactive mastery experiences are the most important source of efficacy information because they provide the most authentic evidence of whether one can muster whatever it takes to succeed…A resilient sense of efficacy requires experience in overcoming obstacles through perseverant effort…The relative power of guided enactive mastery…produces stronger and more generalized efficacy beliefs than do modes of influence relying solely on vicarious experience, cognitive simulations, or verbal instruction.” [4].

### Relevant computational theories

For a system such as DStress, it seems desirable to be able to make precise predictions of the probability of success of a given individual on a given exercise on a given day. More generally, mHealth interventions are expected to operate in intensive interactions with individuals, perhaps multiple times per day, and possibly instrumenting behavior in a nearly continuous fashion. By contrast, widely used health behavior theories at the level of the individual [12] involve explanatory constructs related by linear functions to static snapshots of behavioral data taken at much coarser timescales [2]. In general, there have been few attempts to refine goal-setting theory, social cognitive theory, or the theory of planned behavior to develop precise, predictive models of the fine-grained daily dynamics of motivation, self-efficacy, and ultimately goal achievement. It has been argued [2] that such refinement and precision is needed to better support foundational science in behavior change and support new technologies such as mHealth.

Although lacking detail and precision, many of the high-level constructs at the core of current behavior change theory are defined by reference to underlying cognitive processes. For instance, the construct of self-efficacy in social cognitive theory has been defined in terms of an underlying learning process, whether from one’s own experience or by observation of others [4]. Cognitive expectations and evaluations about changing one’s behavior are at the core of the theory of planned behavior and are assumed to be based on past experiences (perceptions and memories) [5]. Implementation intentions are a form of prospective memory [13, 14].

Martín et al. [15] describe a dynamical system model of social cognitive theory, including self-efficacy. Navarro-Barrientos et al. [16] present a similar model of the theory of planned behavior, including PBC. Both of these models are based on a fluid analogy (specifically a fluid inventory control system analogy) in which *inventories* represent quantities of a psychological construct such as self-efficacy, and *inflows* and *outflows* capture relations among components and factors. These models are specified in a set of differential equations that capture the dynamics of the system at the granularity of day-to-day changes (in principle, such models can track changes at smaller or larger timescales). Aspects of the behavior of these models have been explored in simulations. Martín et al. [15] reported the percentage fit of model predictions to average physical activity levels (%*fit* = 49.54 %) and self-reported self-efficacy (%*fit* = 34.95 %) that were promising.

Vancouver and colleagues [17, 18] have proposed a more integrative computational model, implementing aspects of multiple theories. Theirs is an approach drawing upon cybernetic perceptual control theory [19] that has provided concrete dynamical predictions in line with goal setting theory [11], self-efficacy [4], and a discontinuous trade-off between self-efficacy and intensity of effort [6, 7]. Bandura [20, 21] has criticized the perceptual control theory approach on a number of grounds, including the lack of internal self-representation and lack of rich cognitive habits and skills for self-regulation (or self-disruption) ([20, 22], pp 22–23).

### Motivations for applying ACT-R

The predictive computational models of self-efficacy in both Martín et al. [15] and Vancouver et al. [18] are variations on control theory, which is often used to understand and control (e.g., optimize) complex dynamical systems whose input-output behavior is modified by feedback. In contrast to these control-theoretic approaches, I propose that the high-level constructs found in social cognitive theory and other behavior change theories can be refined to be more precise and dynamical via ACT-R [9], which specifies the time course of cognition and learning in reaction to moment-by-moment interactions with the environment. ACT-R also supports a more complete (and psychologically plausible) range of cognitive representation and processing capabilities and richer learning capabilities than basic control theories [9].

ACT-R [9, 10] is a unified theory of how the structure and dynamics of the brain give rise to the functioning of the mind. The ACT-R simulation environment is a computational neurocognitive architecture that specifies the theory and supports the development of specific models of individuals. As a unified theory of cognition, ACT-R has been used to explain a wide range of psychological processes [23]. The theory is constrained not only by tests against behavioral data but also by physiological data about underlying neural processing (i.e., from fMRI studies [9]) and mathematical analysis of the adaptive fitness of ACT-R mechanisms given the evolutionary ecology of human cognition [22]. The use of ACT-R to address behavior change is also motivated by the theory’s previous successes in developing applications—particularly in the development of automated intelligent tutoring systems [24–27]. By using the ACT-R simulation architecture, we immediately gain purchase on theoretical mechanisms and constraints that have been studied and refined by a large community of psychological scientists and an established framework for integrated multi-timescale and multi-module explanation.

These motivations for using ACT-R to refine and develop predictive theories of behavior change can be restated as four theses articulated in Newell [23] and Anderson [28]: (a) the *integration thesis*, that neurocognitive architectures provide a unified account of how the modules of the mind function together to produce coherent behavior and provide a basis for an integrative explanation of data produced across specialized domains of psychology [23]; (b) the *decomposition thesis* that longer term behavior change occurring over days, weeks, or months can be decomposed to learning events occupying much briefer units of time and involving smaller chunks of belief, experience, or knowledge; (c) the *modeling thesis* that models in neurocognitive architectures provides a basis for bridging the events at the small scale to the dynamics of behavior change occurring at the large scale; and (d) the *relevance thesis*, that longer term changes and outcomes can be improved by modeling, predicting, and intervening in behavioral events that are occurring at the smaller timescales.

### Summary of the DStress study

Konrad et al. [3] describe an experiment investigating the effects of adaptive (individualized) daily goal assignments. Adult participants (*N* = 65; 19–59 years) were randomly assigned to three conditions with different 28-day goal progressions: (1) a *DStress-adaptive* (*N* = 19) condition using the adaptive coaching system in which goal difficulties adjusted to the user based on past performance, (2) an *easy-fixed* (*N* = 24) condition in which the difficulty of daily goals increased at the same slow rate for all participants assigned to that condition, and (3) a *difficult-fixed* (*N* = 22) condition in which the goal difficulties increased at a greater rate. Konrad et al. [3] found that the adaptive DStress-adaptive condition produced significant reductions in self-reported stress levels compared to the easy-fixed and difficult-fixed goal schedules. Here, the focus is on the success rates in performing assigned daily goals.

### A measurement model for exercise difficulty levels in DStress adherence

The ACT-R neurocognitive model uses estimates of exercise difficulties that are measured from the success (or failure) of DStress participants in executing the specific exercises given as goals. These difficulty estimates were obtained through the application of a Rasch measurement model [29, 30] to the DStress data presented in Konrad et al. [3]. Rasch models are frequently used in the analysis of responses to psychometric or educational test items in order to understand latent (unobserved) properties of the test items and characteristics of the individuals.^1^ Rasch models have also been extended to learning data from computer tutoring systems [31]. Rasch models, when applicable, have a property called *specific objectivity*, which implies, in the DStress case, that the scaling of difficulty levels of different exercises within the given frame of reference is independent of the participants used in estimation.

The difficulty, *δ*_*j*_, of each exercise, *j*, was empirically estimated post hoc from the compliance data using a Rasch measurement model [29, 30, 32]. For the exercise difficulty analysis, the performance of any assigned exercise goal on any given day is coded as a dichotomous variable (“success” = 1 and “failure” = 0), and the model is

1 $$ Pr\left({X}_{ij}=1\right)=\frac{exp\kern0.28em \left({\theta}_i-{\delta}_j\right)}{1+ exp\left({\theta}_i-{\delta}_j\right)} $$

where *X*_*ij*_ is the success/failure of person *i* on exercise *j*, *θ*_*i*_ is a general *ability* parameter estimate for the person, and *δ*_*j*_ is the exercise *difficulty* estimate. In this formulation, the ability and difficulty parameters take on any real values and are measured on the same logit (log odds) scale. Equation 1 is similar to the kinds of models that are often fit to data using ordinary least squares logistic regression; however, the parameter estimation for the Rasch model uses a generalized linear mixed model in which the participant ability parameters are treated as random coefficients [33].

**Fig. 2:**
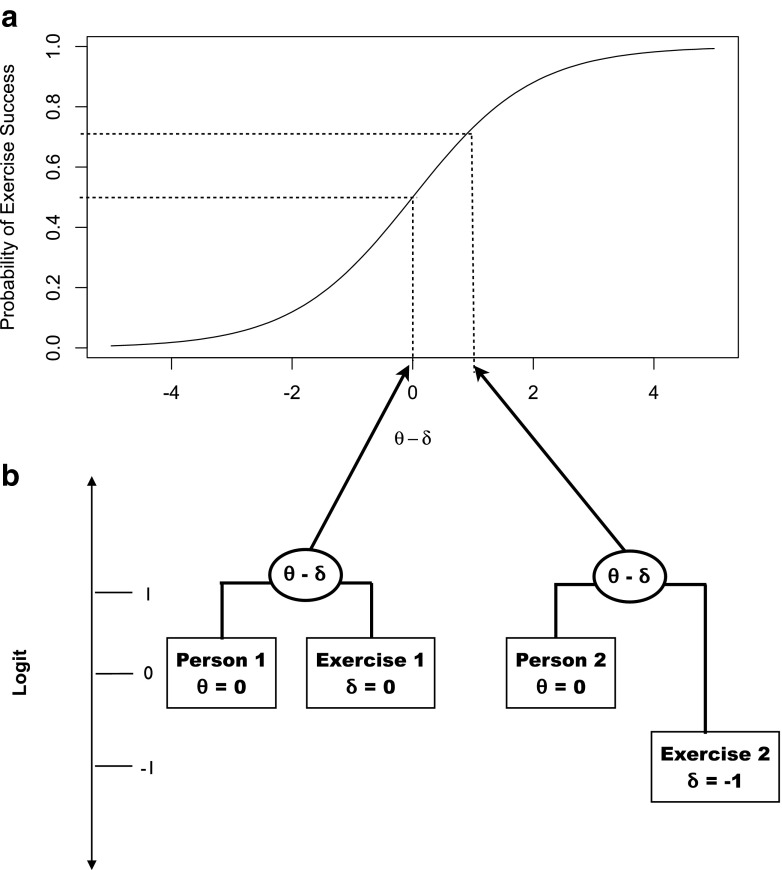
The Rasch measurement model for the DStress data assumes that (**a**) the probability of exercise success is a logistic function of the difference between an individual’s ability and the difficulty of the exercise (*θ* − *δ*) and (**b**) that the individual ability parameters (*θ*) and specific exercise difficulty parameters (*δ*) can be located on a continuous logit (log odds) scale

Figure 2 provides a graphical visualization of the measurement model. Figure 2a shows how the difference between person ability and exercise difficulty, *θ*_*i*_ − *δ*_*j*_, is scored on a logit scale (*x*-axis) and is related to the probability of success Pr(*X*_*ij*_ = 1) as a logistic function. In Fig. 2b, the logit scale is on the *y*-axis and the location on that scale of specific person ability scores, and specific exercise scores, is indicated by boxes (i.e., boxes with scores of 0 are higher up than boxes with scores of −1). To predict the probability of success of Person 1 on Exercise 1, one subtracts *θ* − *δ* (left oval in Fig. 2b), which yields zero, which can then be located on the *x*-axis of Fig. 2a (left arrow in Fig. 2b) and mapped onto a prediction of a probability of success of 0.5 (dotted line in Fig. 2a). By similar graphical reasoning on the right side of Fig. 2, one can predict that Person 2 on Exercise 2 will have a probability of success of 0.73. In general, the probability of success Pr(*X*_*ij*_ = 1) = 0.5 when ability is equal to difficulty, *θ* = *δ*; the Pr(*X*_*ij*_ = 1) > 0.5 when ability is greater than the difficulty, *θ > δ*; and the Pr(*X*_*ij*_ = 1) < 0.5 when the ability is less than the difficulty, *θ < δ.*

**Fig. 3:**
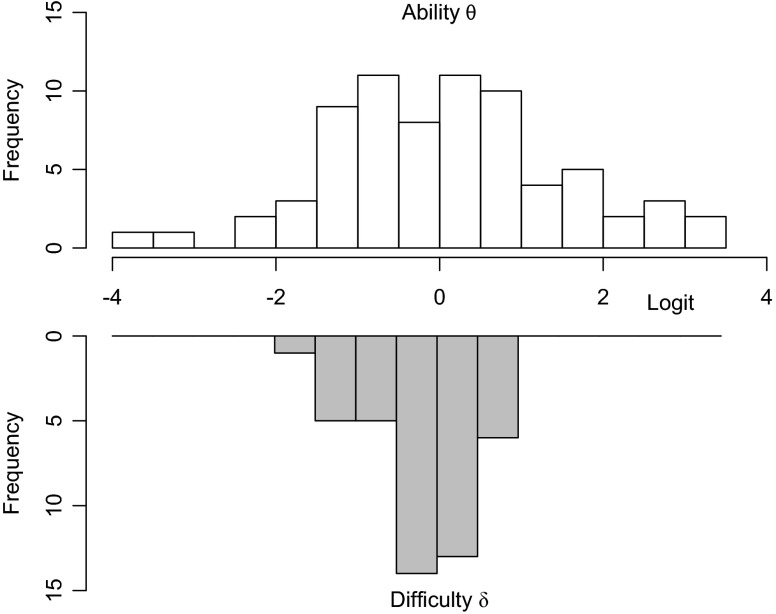
Histogram distribution of the person ability and exercise difficulty parameters in the DStress dataset

**Fig. 4:**
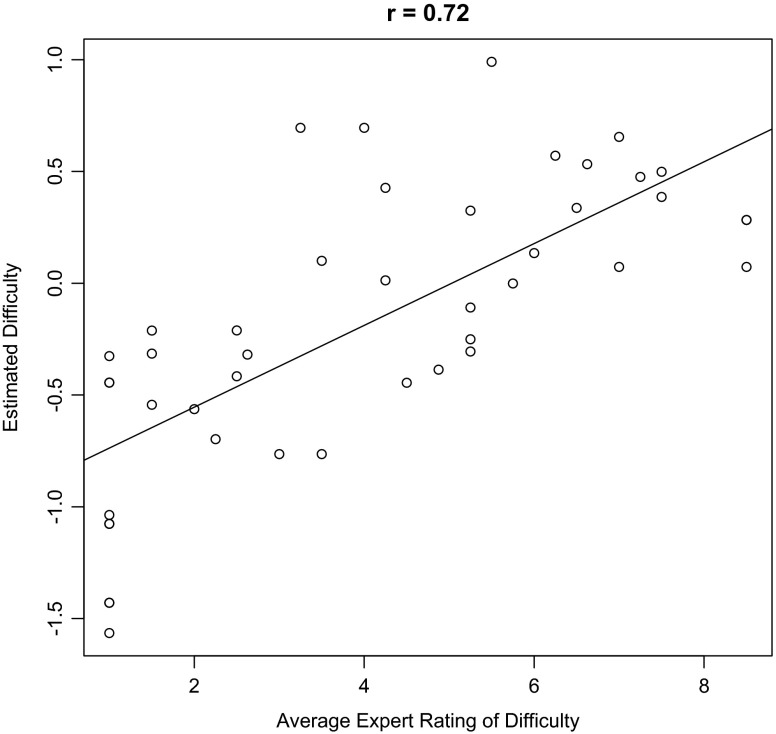
Scatterplot of the exercise difficulty estimates from the Rasch measurement model against expert ratings (10-point scale) obtained in the DStress study, plus best-fit regression line

Figure 3 presents histograms of the person ability parameters and exercise difficulty parameters estimated from the daily DStress exercise adherence data in Konrad et al. [3]. Note that the individual ability scores are approximately normally distributed and centered on zero, whereas the difficulty scores are slightly skewed to be less than zero (*δ* median = −0.21)—i.e., easy enough for the average participant to complete with greater than 50 % success. Figure 4 shows the correlation of the estimated exercise difficulties against the average difficulty ratings (10-point scale) of three experts in Konrad et al. [3].

**Fig. 5:**
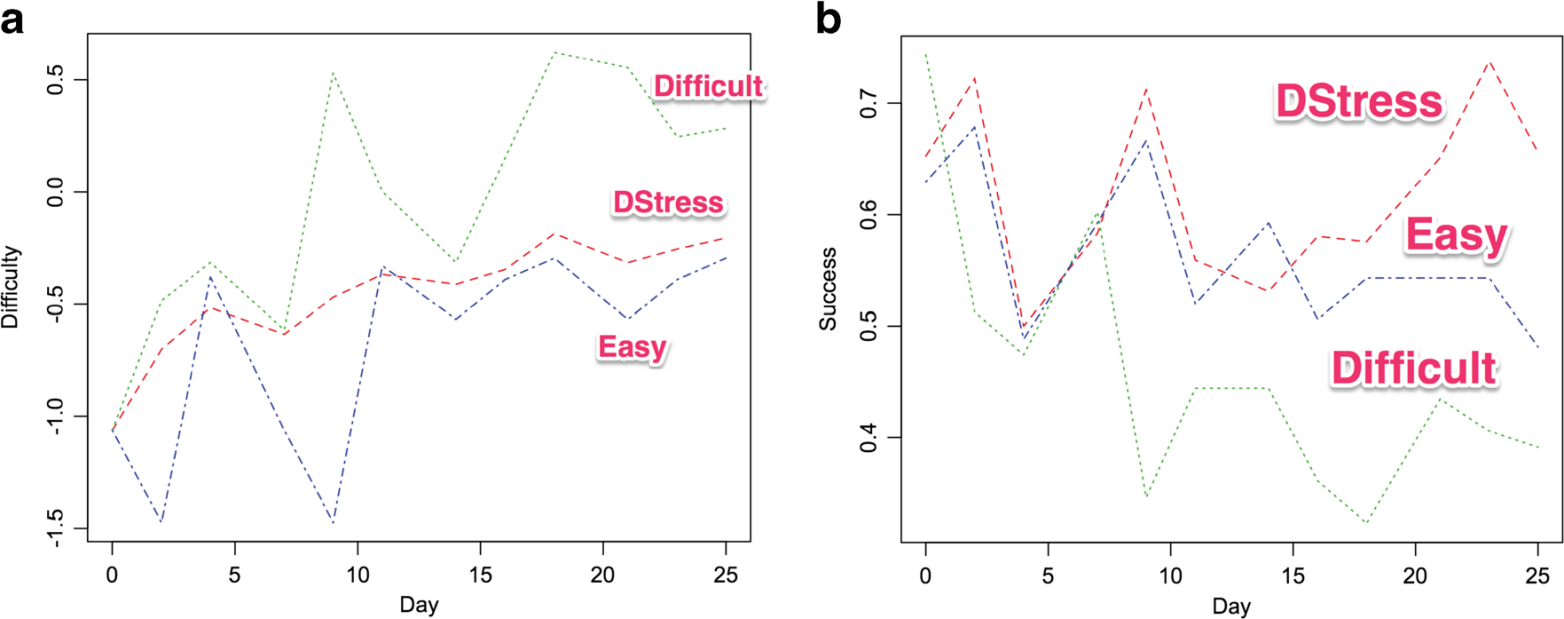
Summary data from Konrad et al. [3]: (**a**) estimated mean daily difficulty of exercises and (**b**) the mean rate of successfully completing assigned exercises

Figure 5a presents an analysis showing how the manipulations in Konrad et al. [3] produced different ramp-ups in exercise difficulty, as was intended. The daily average difficulties (in logits) in Fig. 5a average over the exercise difficulties actually assigned to participants on each day in each condition. Notably, the DStress-adjustable exercise difficulties remain mostly between the levels assigned in the easy-fixed and difficult-fixed conditions.

Figure 5b shows the corresponding success of participants in adherence to the assigned goals. As might be expected, the DStress-adjustable participants having easier exercises than the difficult-fixed condition participants show higher success rates. Notably, by the end of the 28-day programs, the DStress-adjustable participants showed higher adherence rates than the easy-fixed participants, despite being assigned more difficult exercise goals. This increased ability to tackle more difficult goals is consistent with a buildup in self-efficacy through guided enactive mastery.

### ACT-R-DStress

A key aspect of the ACT-R-DStress model is a mechanistic account of how self-efficacy is “perceived.” This “perception” is fundamentally a memory process in which past experiences of efficacy at behaviors similar to a target goal are retrieved and blended together to produce a self-efficacy assessment. This self-efficacy assessment, in turn, is used to set intended effort levels, and self-efficacy and intended effort together determines whether the target behavior will be attempted. Consequently, we should expect that the dynamics of self-efficacy and performance should exhibit the dynamics of the underlying memory mechanisms and exhibit well-known memory phenomena.

### ACT-R

ACT-R [9, 10] is a unified theory of how the structure and dynamics of the brain give rise to the functioning of the mind. The ACT-R simulation environment is a computational neurocognitive architecture that specifies the theory and supports the development of specific models. Models are initialized by detailing the knowledge (or experience) within the system, and the system can learn through interaction with the environment (usually also simulated).

The ACT-R architecture is composed of *modules*, processing different kinds of content, which are integrated and coordinated through a centralized *production module*. Each module corresponds to a brain region: Processing predicted in a specific ACT-R module is a prediction of a correlated activation pattern in a specific brain region. Each module is assumed to access and deposit information into *buffers* associated with the module, and the central production system can only respond to the contents of the buffers, not the internal encapsulated workings of the modules. For instance, the *visual module* is correlated with the occipital cortex (and others) and the *visual buffer* with the parietal cortex. The visual buffer acts as a link (pathway) between the visual module and the production module.

The ACT-R-DStress simulation involves the following modules and buffers:

- *Production module* (basal ganglia), which matches the contents of other module buffers and coordinates their activity.
- *Goal buffer* (dorsolateral prefrontal cortex), which keeps track of the goals and internal state of the system. The goal buffer stores and retrieves information that represents the internal intention of the system and provides local coherence to behavior.
- *Declarative module* (temporal lobe; hippocampus), *retrieval buffer*, and *blending buffer* (ventrolateral prefrontal cortex), associated with the retrieval of knowledge and past experiences from long-term declarative memory. The information in the declarative memory module corresponds to personal episodic and semantic knowledge that promotes long-term coherence in behavior.
- *Imaginal module* (posterior parietal cortex), which serves as a mental scratchpad for temporary memory.

Knowledge is specified in the production module as *production rules*. A production rule can be thought of as a formal specification of the *flow of information* from buffers in the cortex to the basal ganglia and back again. In general, multiple production rules can apply at any point in time, but only one production may execute. Productions have a *utility* property that is used to select the single rule that is executed. Knowledge in the declarative, goal, and imaginal modules (and other modules in ACT-R) is represented formally in terms of *chunks* [34, 35]. A chunk represents a cognitive unit of information encoding a collection of elements—for instance, the elements of an experienced event or the components making up a fact (e.g., the numbers in a friend’s telephone number). Each module is limited to placing a single chunk in a buffer. Chunks have *activation* levels that determine how chunks are retrieved.

Production utilities and chunk activations are real-valued quantities produced by *subsymbolic mechanisms* in ACT-R. These subsymbolic mechanisms reflect neural-like processes that determine the time course and probability of cognitive activity and behavioral performance. The dynamics of declarative memory retrieval and production selection are determined by these subsymbolic mechanisms.

The following is a simulation printout of a memory chunk used in the ACT-R-DStress simulation:

BEHAVIOR-EXPERIENCE100-0

ISA BEHAVIOR-EXPERIENCE

BEHAVIOR MARCHING_IN_PLACE

DIFFICULTY −0.013206851

ABILITY 0.025988732

EFFORT 0.242358

OUTCOME SUCCESS

The first line is just a name used for convenience to identify the chunk. Each subsequent line contains a *slot* with a *value*. The *isa* slot indicates that this chunk is a type of *behavior experience*, and the following *behavior* slot indicates that the experience was for the “marching in place” exercise. The difficulty, ability, and effort slots contain values that link to subjective somatic experiences discussed below. The final *outcome* slot indicates that in the case of this particular experience, the exercise was performed successfully.

The following is a simulation printout of a production rule (with some nonessential elements omitted):

(p request-perceived-ability

=goal>

isa behavior-goal

difficulty =difficulty

behavior =behavior

ability nil

==>

+blending>

isa behavior-experience

behavior =behavior

outcome success)

The first line is just a convenient name used for identification. The next five lines before the “==>” arrow are the *conditions* of the production rule that must match the current state of the ACT-R buffers. The labels proceeded by an equal sign, such as =*behavior*, are variables and can match arbitrary symbols in a buffer. In this case, the conditions specify a match to a chunk in the goal buffer (second line), which is a *behavior goal* to do a *behavior* for which the *ability* slot (self-efficacy) has not been assessed (is “nil”). The lines following the ==> arrow are the *actions* of the production. In this production, the action is to try to retrieve from memory (using *blending*, discussed below) information about *behavior experiences* similar to the goal behavior. In essence, the rule says, “IF my goal is to do an exercise, and I have not assessed my self-efficacy THEN try to remember similar exercises on which I was successful.”

### Somatic markers

A recent extension [36] of ACT-R is the incorporation of *somatic markers* [37] that encode associations among experiential chunks to physiological affective states (“bodily feelings”). These somatic markers, when associated with experiences of various outcomes of prior responses, produce emotional reactions when a person faces situations or decisions that invoke recall of those past experiences. In the ACT-R-DStress model, I assume that a variation of somatic markers represent remembrances of efficacy at past performances (i.e., how difficult something was to do) and motivational effort experiences (i.e., how much effort was it necessary to put in). This assumption is also consistent with those of Kukla’s [6] attributional theory of performance.

### Declarative memory and blended retrieval

Table 1 presents a subset of the ACT-R subsymbolic mechanisms that are relevant to the current ACT-R-DStress model. These key mechanisms are involved in the strengthening of declarative memories for repeated experiences and the processes of memory retrieval and blending. The *probability of retrieval* of a memory chunk is dependent on the current *activation level* of that chunk in comparison to the summed activation of all chunks. The activation values, in turn, are dependent on a *base-level learning* mechanism that captures the history of experience for each chunk. Each experience involving a chunk produces a gain of activation that decays as a power function of time, and multiple experiences produce a summation of activation impulses. This produces a *power law of forgetting*: The success and speed of retrieving memories decline in a lawful way with time (a *lag effect*). Base-level learning also yields a *power law of practice*: Success and speed of memory retrieval improve in a lawful way with repetition (a *frequency effect*).

**Table 1:** Key subsymbolic memory blending mechanisms in ACT-R-D stress

Mechanism	Equation	Description
Blended retrieval	$V= \min {\displaystyle \sum_i{P}_i}{\left(1-Sim\left(V,{V}_i\right)\right)}^2$	*P*_*i*_ *:* Probability of declarative retrieval *Sim*(*V, V*_*i*_) Similarity between compromise value *V* and retrieved value *V*_*i*_
Retrieval probability	${P}_i=\frac{e^{A_i/s}}{{\displaystyle {\sum}_j{e}^{A_j/s}}}$	*P*_*i*_: The probability that chunk *i* will be recalled *A*_*i*_: Activation strength of chunk *i* *∑A*_*j*_ *:* Activation strength of all of eligible chunks *j* s: Chunk activation noise
Activation	*A*_*i*_ = *B*_*i*_ + *ε*_*i*_	*B*_*i*_ *:* Base-level activation reflects the recency and frequency of use of chunk *i* ε_*i*_: Random noise value
Base-level learning	${B}_i=ln\left(\sum_{j=1}^n{t}_j^{-d}\right)+{\beta}_i$	*n*: The number of experiences for chunk *i* *t*_*j*_: The time since the *j*th presentation *d*: A decay rate *β*_*i*_: A constant offset

Blending [38] is an extension of declarative memory retrieval in which values from multiple retrieved chunks are combined to produce a compromise value. Each experiential chunk in memory may encode some specific value, *V*_*i *_—for instance, the difficulty of a specific exercise experience. A blended memory retrieval of the difficulty of past experiences would weigh the contribution of each memory by the probability of retrieval of each chunk. The compromise value is also determined by a psychological similarity function *Sim*(*V*, *V*_*i*_) between the compromise value and the specific values encoded in memory chunks.

As experiences of overcoming more difficult goals are added to memory, those experiences will alter the blended self-efficacy value to become greater. The frequency, recency, and difficulty of those experiences have effects on the final blended value of self-efficacy, because of the mechanisms of base-level learning.

### Process model

In outline, the model involves the following steps:

- A *behavioral goal* is considered for doing one or more *activities* that are believed to have some level of *difficulty*, *δ*, to being performed.
- *Blending an assessment of self-efficacy*. A *blended retrieval process* is initiated in long-term *declarative memory* to recall successful experiences involving activities *similar* to the behavioral goal activities. This process blends the difficulty levels of those past experiences into a composite assessment of the difficulty levels achieved in past (similar, recalled) experiences, and this is mapped directly to set an assessment of *self-efficacy*, *θ*, for the behavioral goal. So, self-efficacy is set by remembering the difficulties overcome in the past on activities similar to the goal.
- *Blending an intended effort* 2 *level*. A blended retrieval process is initiated to recall past experiences of success with similar levels of perceived self-efficacy and perceived activity difficulty for the goal. This process blends an assessment of intended effort levels, *ψ*, which had been required to achieve success in those past experiences. In general, higher levels of intended effort will be recalled for experiences in which the difficulties overcome were well beyond the levels of self-efficacy at the time.
- *Predicting success*. Based on the goal difficulty, *δ*, perceived self-efficacy, *θ*, and intended effort, *ψ*, the model makes a prediction about the likelihood of success.
- *Doing it*. If the expected probability of success is above a threshold, it is attempted.
- *Storing the new experience*. If the activity is attempted, the experience is stored in memory and influences future attempts.

The model makes the strong assumption that the psychological prediction of performance can be characterized as a subsymbolic computation of the log odds (logit) of success as

2 $$ \log it(s)=\theta +\varPsi -\delta $$

where *s* is the probability of successful performance. So, predicted performance success increases with self-efficacy and intended effort and decreases with activity difficulty. This log odds formulation is consistent with other ACT-R subsymbolic mechanisms (i.e., activation) and consistent with measurement approaches that yield real-valued empirical quantities [31].

### Model-tracing the daily adherence of an individual

The individual-level predictions of the model were compared to the DStress data using a model-tracing approach [27].

To model-trace each participant in the dataset:

1. Initialize task knowledge about behavioral goals and exercises
2. Initialize a hypothetical set of background experiences about past behavioral attempts that is consistent with pre-experimental survey measures of self-efficacy.
3. For each exercise in each session on each day
  - Set the exercise goal with difficulty, *δ*, corresponding to the next assigned exercise goal in the dataset
  - Blend a memory-based assessment of self-efficacy for target goal, *θ*
  - Blend a memory-based assessment of intended effort, *ψ*
  - Predict likelihood of success, *s*
  - Compare the predicted success to the observed report of success
  - Store the observed experience in memory

The task knowledge in step 1 is common to all the simulations of participants. The initialization of background experiences in step 2 is generated to span abilities from −1.0 to +1.0 in increments of 0.1, five levels of exercise difficulties from −1.5 to 0.5, and two levels of intended effort (0, 0.25). These values were selected so that range of abilities was balanced around zero and the difficulties were balanced around −0.5 to approximate the distributions in Fig. 3. The intended effort levels were arbitrary choices (assumed to be bounded below by zero). Small deviations from these ranges appear to have no major effect on the model results. Ten background chunks for each combination of levels of ability, difficulty, and effort were generated, and the success or failure associated with that experience was determined by a probability dependent on ability, difficulty, and intended effort (i.e., the logit equation above).

The loop specified in step 3 iterates through the dataset. The goal difficulties of each exercise were set using the parameter estimates provided by the Rasch model. On each iteration, the simulation produced a prediction of success that was compared to the observed report. Each observed success or failure is stored as a new behavioral experience in ACT-R-DStress memory.

## RESULTS

### DStress adherence exhibits core memory phenomena predicted by ACT-R

**Fig. 6:**
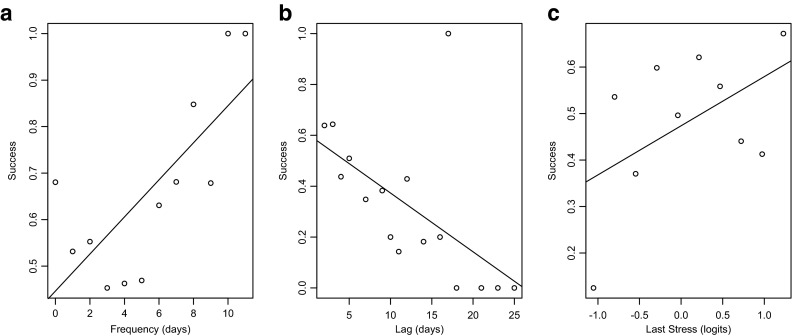
Mean success rate of daily exercise adherence as a function of (**a**) frequency of days of past success, (**b**) lag days since last success, and (**c**) “stress” increment of the difficulty of assigned exercise goal over past exercise difficulties achieved. Each data point in each graph averages over all the success data available from all participants for the particular levels plotted on the *x*-axes

Figure 6 re-plots the exercise adherence data in Fig. 5b to present evidence for memory-like phenomena underlying success. Figure 6a shows that the mean rate of success increases as a function of the frequency of past successes on previous days’ exercise goals. This is consistent with the frequency effects predicted by base-level learning. Figure 6b shows that the success rates decrease as a function of the lag in days since the last achievement of an exercise goal. This is consistent with the lag effects predicted by base-level learning. Figure 6c shows that the rate of success increases as a function of the *stress* of the exercises performed on the last day of successfully achieving goals. This stress is the increment in difficulty of the attempted exercises over previously attempted exercises. The improvement of success with size of stress is consistent with the blending mechanism.

### DStress model fit

**Fig. 7:**
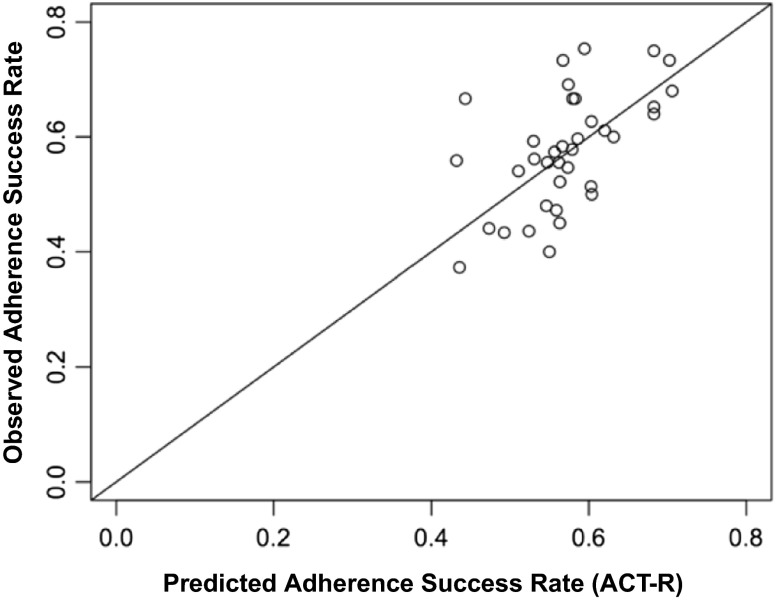
Observed exercise adherence success as a function of the ACT-R-DStress prediction. Each data point is averaged over observations and model predictions pooled by group condition and day

Figure 7 presents a comparison of the ACT-R-DStress simulation predictions produced in model-tracing (described above) and the observed data summarized in Fig. 5b. The model produces a predicted success or failure for each and every exercise on every exercise day for every participant. Each point in Fig. 7 pools the observed and predicted success data by day and by condition (DStress-adaptive, easy-fixed, difficult-fixed). On any “exercise day,” there were three exercise goals assigned to each individual in each of the three conditions, so the observed success rate for a particular condition on that day is the proportion of successful exercise completions by all individuals in that condition divided by the total number of exercises assigned to those individuals. The predicted success rate is similarly computed but uses model-predicted success/(success + failures) rather than the observed. ACT-R parameter settings were not systematically explored and were set in a way consistent with other ACT-R models [Lebiere, personal communication]. In other words, no free parameters were estimated or explored to produce the model fits displayed in Fig. 7. The code for the ACT-R-DStress model along with the parameter settings is available in the Supplemental Materials for this article. For these averaged points, *RMSE* = 0.083.

### Mathematical model

In many applications, it has been useful to develop mathematical models that approximate the predictions of ACT-R. This obviates the need to actually run complete ACT-R simulations in order to make useful predictions that guide interventions with individual people. For instance, intelligent tutoring systems based on earlier versions of the ACT theory successfully employed student models that were based on mathematical models of cognitive skill acquisition but ignored (for instance) modeling the details of declarative memory predictions [27]. Another example is optimal scheduling of vocabulary memorization [39] for second-language learning that uses a mathematical model of base-level learning in declarative memory (see Table 1). Here, I present a mathematical model that captures key aspects of base-level learning and blending in declarative memory.

The model captures the three key signature phenomena presented in Fig. 7: (a) frequency, (b) lag, and (c) stress (the difference in difficulty between the best achievement so far and the difficulty of a newly achieved goal). The ACT-R mechanisms that give rise to these phenomena are base-level learning (for frequency and lag) and blending (for stress).

The gains in self-efficacy, ς, over the course of *t* days, 1…*t*, of behavior change attempts can be represented as

3 $$ \varsigma (t)={\displaystyle {\sum}_{k=1}^t\left[{\left(t-k\right)}^{-d}\left({\beta}_1+\gamma {\varDelta}_k\right)\right]} $$

where *d* is the memory decay parameter, γ is a gain parameter, *β*_1_ is an offset parameter, and Δ_*k*_ is the stress of goal *k*,

4 $$ {\varDelta}_k={\overline{\delta}}_k-{\overline{\delta}}_m $$

where ${\overline{\delta}}_k$ is the average difficulty of activities on a particular day *k* and ${\overline{\delta}}_m$ is the average difficulty of the activities on the last successful day prior to *k*. In the model fit below, these difficulties are just based on the ones estimated by the Rasch model earlier (Eq. 1).

To predict the success, *X*_*ij*_, of person *i* on behavior goal *j* at time *t*, I incorporate the self-efficacy gain Eq. 3 into the following:

5 $$ Pr\left({X}_{ij}=1\Big|{\theta}_i,{a}_i,{\delta}_j,t\right)=\frac{exp\left({\beta}_0+{\theta}_i-{\delta}_j+{a}_it{-}^d+\varsigma (t)\right)}{1+ exp\left({\beta}_0+{\theta}_i-{\delta}_j+{a}_it{-}^d+\varsigma (t)\right)} $$

where *θ*_*i*_ and *δ*_*j*_ are the individual abilities and goal difficulties estimated above and *β*_0_ is an intercept parameter. The term *α*_*i*_ *t* ^− *d*^ is intended to represent a pre-program initial impulse of intention to change that decays as a power function of time. As is turns out, the model-fits suggest that it is zero in the data.

**Fig. 8:**
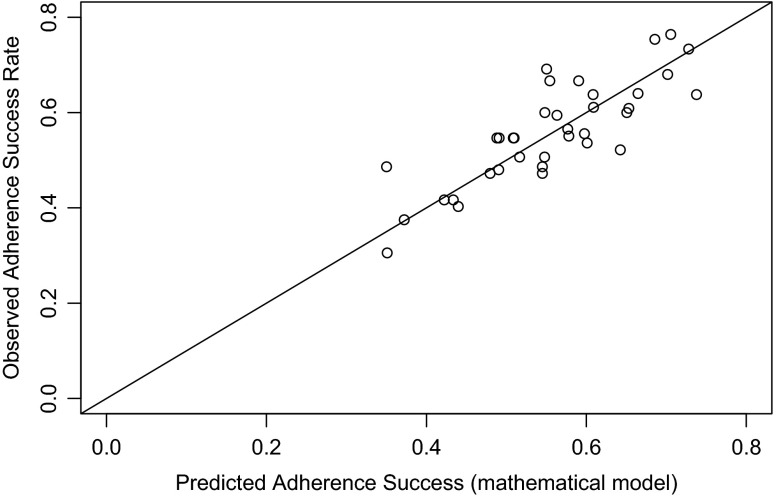
Observed exercise adherence success as a function of the mathematical model prediction. Each data point is averaged over observations and model predictions pooled by condition and day

Equation 5 was fit to the DStress data using the *port* nonlinear least squares algorithm [40] computed in the R *nls* function. The fit of the model to the data is presented in Fig. 8. Comparison of Figs. 8 to 7 suggests an improved fit by the mathematical model, which is to be expected given that the parameters have been estimated to provide a best fit to the data. The *RMSE* = 0.060, which is indeed better than that for the ACT-R-DStress simulation. The parameter estimates are *β*_0_ = 0.1223, 95 % CI [−0.1737, 0.2552], *β*_1_ = -0.1790, 95 % CI [−0.4461, 0.0578], *γ* = 0.2312, 95 % CI [**0**, 0.5704], *d* = 0.9394, 95 % CI [0.1204, **1**], and as noted above, *α* = 0.0, 95 % CI [**1**, 0.2390].^3^

### General discussion and implications

**Fig. 9:**
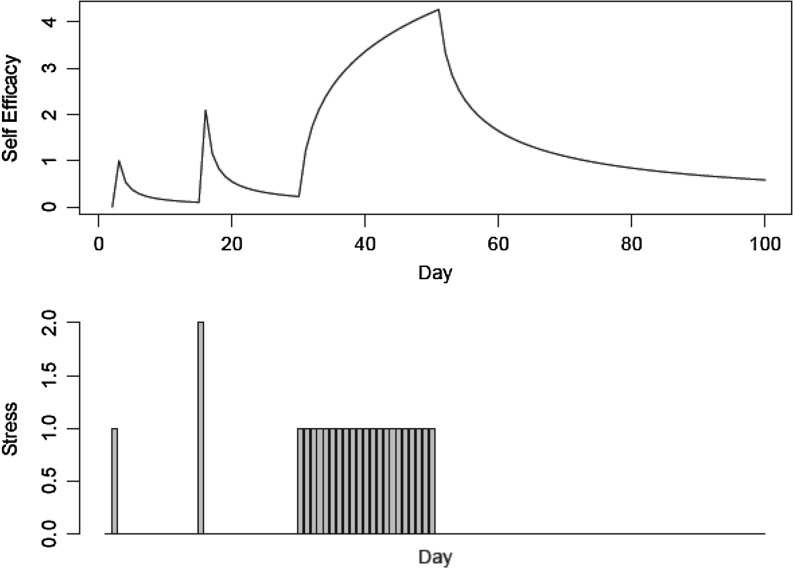
A hypothetical set of successful goal achievements over days for activities at different levels of stress (the *two leftmost bars*) and different levels of inter-day lag (*bottom*). The resultant gains and decays of self-efficacy given those achievements (*top*). All scales are arbitrary

Figure 9 presents a visualization that illustrates the basic dynamics of ACT-R-DStress and the associated mathematical model. The models are essentially “impulse” models in which each impulse decays with time, and impulses add to prior ones. Larger degrees of stress produce bigger impulses, and positive impulses at high frequency and low lags build up rapidly. This intuition meshes with the general notion that positive experiences at behavior change build up self-efficacy, but those can decay with time, and substantial achievements produce bigger boosts in self-efficacy.

It is worth noting that this impulse model of self-efficacy is very similar in spirit to the Banister impulse-response training model [41] proposed for optimizing physical training schedules for athletes. Whereas the Banister model is aimed at predicting physical changes, the ACT-R models presented here are aimed at predicting psychological changes. But, the form and dynamics of these two sets of models are virtually the same. Perhaps the mind and body are actually very much in harmony.

The aim of this paper was to present an explanatory and predictive model of behavior change success in an mHealth study that was specifically aimed at improving self-efficacy through personalized guided enactive mastery. The success of this modeling suggests that fine-grained dynamics of other psycho-social aspects of behavior change might be fruitfully addressed by models developed in neurocognitive architectures. Collectively, such models could provide a basis for user models that drive personalized mHealth interactions that increase engagement and success in behavior change.

The specific ACT-R-DStress model illuminates the four broad theses presented in the introduction. The modeling of goal, memory, learning, and performance mechanisms to provide a cohesive account of self-efficacy, guided enactive mastery, and adherence is consistent with the integration thesis. Broad gains in exercise behavior over the course of 28 days can be refined into finer-grained goal striving and memory events happening within a day, which is consistent with the decomposition thesis. The ACT-R architecture provides a theoretical account that explains and predicts the linkage of the small-scale events to the large-scale phenomena, in line with the modeling thesis. What remains is to demonstrate the relevance thesis, which would involve using the computational predictions to interact with individuals in ways that optimize their success at behavior change.

## Footnotes

1 The Rasch model used here is sometimes referred to as a *one-parameter logistic* (1PL) model as it has just one parameter for the difficulty of test items. Item response theory models in psychometrics may have multiple parameters (e.g., 2PL or 3PL models are common) or have multidimensional item parameters.

2 Vancouver et al. (2008) use this same construct but call it “motivation.” Throughout, I revert to the original name “intended effort” used in Kukla (1972) for this construct.

3 CI values in **bold** for *γ*, *d*, and α indicate an upper or lower bound constraint in the mathematical model that was used by the nonlinear least squares *port* algorithm—for instance, the decay, *d*, is constrained to be less than 1.0.
